# Serological Evidence of Exposure to Onyong-Nyong and Chikungunya Viruses in Febrile Patients of Rural Taita-Taveta County and Urban Kibera Informal Settlement in Nairobi, Kenya

**DOI:** 10.3390/v14061286

**Published:** 2022-06-13

**Authors:** Moses Muia Masika, Essi M. Korhonen, Teemu Smura, Ruut Uusitalo, Joseph Ogola, Dufton Mwaengo, Anne J. Jääskeläinen, Hussein Alburkat, Yong-Dae Gwon, Magnus Evander, Omu Anzala, Olli Vapalahti, Eili Huhtamo

**Affiliations:** 1 KAVI Institute of Clinical Research, University of Nairobi, POB 19676, Nairobi 00202, Kenya; ogolajoseph2000@gmail.com (J.O.); oanzala@kaviuon.org (O.A.); 2 Department of Medical Microbiology, University of Nairobi, POB 19676, Nairobi 00202, Kenya; dmwaengo@yahoo.com; 3 Department of Virology, University of Helsinki, 00014 Helsinki, Finland; essi.m.korhonen@helsinki.fi (E.M.K.); teemu.smura@helsinki.fi (T.S.); ruut.uusitalo@helsinki.fi (R.U.); anne.jaaskelainen@helsinki.fi (A.J.J.); hussein.alburkat@helsinki.fi (H.A.); olli.vapalahti@helsinki.fi (O.V.); eili.huhtamo@helsinki.fi (E.H.); 4 Department of Veterinary Biosciences, University of Helsinki, 00014 Helsinki, Finland; 5 HUS Diagnostic Center, HUSLAB, Virology and Immunology, Helsinki University Hospital, 00029 Helsinki, Finland; 6 Department of Geosciences and Geography, University of Helsinki, 00014 Helsinki, Finland; 7 Department of Clinical Microbiology, Umeå University, 90185 SE Umeå, Sweden; kwon.yongdae@umu.se (Y.-D.G.); magnus.evander@umu.se (M.E.)

**Keywords:** arbovirus, alphavirus, chikungunya, Onyong-nyong, seroprevalence, ELISA, neutralization, immunofluorescence, febrile illness

## Abstract

Several alphaviruses, such as chikungunya (CHIKV) and Onyong-nyong (ONNV), are endemic in Kenya and often cause outbreaks in different parts of the country. We assessed the seroprevalence of alphaviruses in patients with acute febrile illness in two geographically distant areas in Kenya with no previous record of alphavirus outbreaks. Blood samples were collected from febrile patients in health facilities located in the rural Taita-Taveta County in 2016 and urban Kibera informal settlement in Nairobi in 2017 and tested for CHIKV IgG and IgM antibodies using an in-house immunofluorescence assay (IFA) and a commercial ELISA test, respectively. A subset of CHIKV IgG or IgM antibody-positive samples were further analyzed using plaque reduction neutralization tests (PRNT) for CHIKV, ONNV, and Sindbis virus. Out of 537 patients, 4 (0.7%) and 28 (5.2%) had alphavirus IgM and IgG antibodies, respectively, confirmed on PRNT. We show evidence of previous and current exposure to alphaviruses based on serological testing in areas with no recorded history of outbreaks.

## 1. Introduction

Alphaviruses are enveloped RNA viruses and form the only genus in the *Togaviridae* family [1]. In humans, alphaviruses are mainly transmitted through mosquito vectors [2,3]. They are classified into two sub-groups based on where they were first detected. The ‘Old world’ or Eurasian–African–Australasian sub-group, which is associated with fever, rash, and arthralgia includes well-described pathogens, such as chikungunya (CHIKV), Onyong-nyong (ONNV), Sindbis (SINV), and Ross River virus and the ‘New world’ or American sub-group, which is associated with encephalitis, includes Western, Eastern, and Venezuelan equine encephalitis viruses [2,3]. Alphaviruses are also grouped into eight serocomplexes based on antigenic properties. CHIKV and ONNV belong to the Semliki Forest virus complex while SINV belongs to the Western equine encephalitis complex [2,3].

In Kenya, CHIKV and ONNV are the most commonly reported alphaviruses in humans [4,5]. Both viruses are known to exist in sylvatic cycles in Africa but the wild host species are not well defined in Kenya [6]. CHIKV has been causing sporadic outbreaks in Africa for decades and has grown into global prominence over the last two decades becoming a global public health concern [7,8,9]. CHIKV is transmitted by *Aedes (A.) aegypti* and *A. albopictus* mosquitoes. One of the factors that fueled the spread of CHIKV across the globe is a mutation (A226V) in the E1 glycoprotein that enhanced its transmission by *A. albopictus* [10]. This mutation occurred during an outbreak that started in coastal Kenya in 2004 and spread to the Indian Ocean islands, India, and Southeast Asia [9]. Since then, two other CHIKV outbreaks have been documented in Kenya, one in 2016 that occurred in Mandera in the country’s north-eastern tip and another in late 2017 to 2018 in Mombasa, coastal Kenya [11,12]. CHIKV causes fever, severe joint pain, muscle pains, headache, nausea, fatigue, and rash [13], and is transmitted in urban cycles between humans by *Aedes* mosquitoes [2,14]. ONNV was first described in an outbreak that occurred in Uganda in 1959, and thereafter it has been detected in various African countries, including Kenya [4,5]. It is associated with fever, polyarthralgia, rash, and lymphadenopathy, and is clinically indistinguishable from CHIKV [15,16]. ONNV is transmitted by *Anopheles* mosquito vectors [17] and, therefore, tends to occur in areas where malaria is also common [18,19] CHIKV and ONNV are genetically and antigenically closely related. They belong to the Semliki Forest serocomplex and are cross-reactive in serological assays used in laboratory diagnostics [20,21].

SINV was first detected in an eponymous district in northern Egypt in 1952. Its spread is amplified by infected migratory birds flying from northern Europe to Australia. It is transmitted by several mosquito genera including *Culex*, *Culiseta*, and *Aedes*. The virus causes manifestations similar to other arthritogenic alphaviruses, presenting with fever, arthralgia and maculopapular rash [22,23,24]. In Kenya, SINV has been isolated in mosquito vectors but not in humans [25]. A few studies report a low seroprevalence of SINV in areas near the Great Rift Valley lakes and coastal Kenya which are known to be along the flyways for migratory birds [4,24,25].

Several studies show that CHIKV and ONNV are important causes of febrile disease in Kenya [19,26]. The seroprevalence reported for CHIKV and ONNV varies depending on the study population, geographic location and diagnostic assays used. Many of these studies have reported higher alphavirus seroprevalence in western Kenya than elsewhere in the country [4,5,19,27,28,29]. One study that included samples from patients with a febrile rash illness between 2008 and 2014 detected alphavirus (CHIKV or ONNV) antibodies in two individuals in Nairobi (*n* = 179), and one in Taita-Taveta (*n* = 35) [30]. In this study, we aimed to assess the prevalence and previous exposure to CHIKV, ONNV and other alphaviruses in patients with acute febrile illness in rural (Taita-Taveta) and urban (Kibera, Nairobi) locations in Kenya, where limited previous data were available, and where no epidemic activity or outbreaks have been reported.

## 2. Materials and Methods

**Figure 1:**
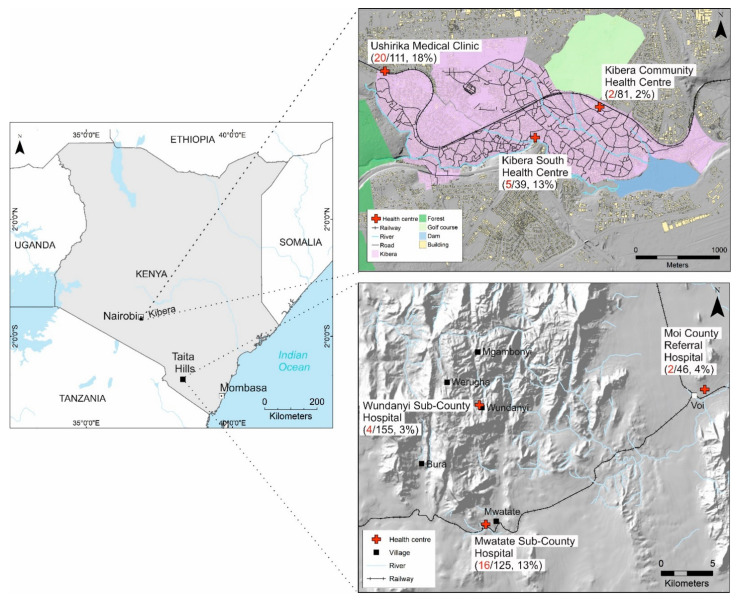
A map of Kenya showing the study sites in Taita-Taveta County and Kibera slum, Nairobi. Indicated alongside the site names is the number of samples per site (denominator) and prevalence of chikungunya IgG antibodies by immunofluorescence assay (red text and percentage). Geographic information system (GIS) data for Kibera was obtained from Kounkuey Design Initiative [34]. Hillshade for Kibera was created in GIS software from digital elevation model (DEM) 5 m and for Taita-Taveta, hillshade was created from DEM 20 m [35].

Study design: This was a cross-sectional study. We analyzed samples that had been previously tested for flavivirus infection [31]. The samples were collected from febrile patients in two geographically disparate regions in Kenya (Figure 1). Taita-Taveta County is located in rural southern Kenya, an ecologically diverse area with a rapidly growing population [32] and little previous research on mosquito-borne viruses in humans [31]. Taita-Taveta County has an elevation range of 400 m in the lowest areas to 2200 m in Taita hills. Kibera slum in Nairobi is the largest informal settlement in Kenya and has a high population density with an estimated 250,000 residents [33]. In Taita-Taveta, serum samples were collected between April and August 2016, and in Kibera slums, plasma samples were collected between February and June 2017. We collected samples and pertinent medical data from patients presenting to six hospitals with acute febrile illness. Samples were stored at −20 °C at hospital labs for one to three weeks before shipment to the KAVI Institute of Clinical Research Lab in Nairobi for storage at −80 °C. All samples were later shipped on dry ice to the University of Helsinki, Finland. A subset of samples was later shipped to Umeå University, Sweden for confirmatory alphavirus plaque reduction neutralization assays.

IgG and IgM antibody testing: Samples were tested for CHIKV IgG antibodies using an in-house IFA modified for CHIKV [36]. Briefly, CHIKV Ross strain-infected Vero E6 cells were detached with trypsin and mixed with uninfected Vero E6 for use as background control. The cells were then washed with phosphate-buffered saline, spotted on glass slides and air dried, fixed with acetone and stored at −70 °C before use. In initial IgG IFA screening, serum or plasma samples were diluted 1:20 in phosphate-buffered saline (PBS), added to pre-spotted slides, and incubated at 37 °C for 30 min in a moist chamber. The slides were washed three times with PBS and incubated at 37 °C for 30 min with FITC-anti-human IgG conjugate (Jackson Immuno Research Laboratories, West Grove, PA, USA) diluted 1:100 in PBS, then washed again three times with PBS and once with distilled water, and examined using a fluorescence microscope. Positive samples were retested as a dilution series in PBS from 1:10 to 1:2560 (1:10, 1:40, 1:160, 1:640, 1:2560). The reciprocal of the last positive dilution was recorded as the IFA IgG titer. All 557 samples were also tested for CHIKV IgM antibodies using a commercial ELISA assay (Chikungunya virus (CHIKV) ELISA incl. IgG/RF absorbent, Euroimmun, Lübeck, Germany) as per the manufacturer’s instructions.

Plaque Reduction Neutralization Tests: A subset of CHIKV IgG and/or IgM seropositive samples (*n* = 58) and eleven IgM/IgG negative samples were tested using plaque reduction neutralization test (PRNT) for CHIKV, ONNV, and SINV antibodies. The 20 seropositive samples were excluded from PRNT due to insufficient volume. The PRNT was based on a method described previously [5]. In brief, Vero B4 (African green monkey kidney) cells were seeded in 96-well plates (15,000 cells/well). Then, 50 μL of threefold serial dilutions of the serum samples (diluted from 1:40, 1:80, 1:160 to 1:320) was mixed with 50 μL of the respective virus (50 pfu), incubated for 1 h at room temperature, thereafter the mixture was transferred to 12-well plates containing a confluent monolayer of Vero B4 cells. After additional incubation for 1 h at 37 °C in a carbon dioxide incubator, the cells were overlaid with 100 μL of Dulbecco’s modified Eagle medium containing 2% carboxymethylcellulose (CMC; Sigma Life Science, St. Louis, MO, USA). After 3 days, the CMC overlay was removed, and the cells were fixed with 4% paraformaldehyde solution before being stained with crystal violet. Visible plaques were counted and the titers are expressed as the reciprocal of the serum dilution that showed 80% of plaque reduction. Samples were considered as PRNT positive if the PRNT titer was >40, positive for ONNV if ONNV titers were at least two-fold higher than CHIKV titers, and CHIKV positive if CHIKV titers were at least four-fold higher than ONNV titers [5]. The threshold was lower for ONNV than CHIKV because of the unique one-way cross-reactivity between CHIKV and ONNV—CHIKV antibodies are more likely to cross-react with ONNV antigens than ONNV antibodies with CHIKV antigens [37]. The virus strains used were CHIKV LR2006_OPY1 (GenBank EU224268.1), ONNV IbH 12628 and SINV Lovanger strain (GenBank KF737350.1).

Virus Isolation: For PCR-positive samples, we attempted virus isolation in Vero E6 (ATCC CRL-1586) and C6/36 *A. albopictus* cells (ATCC CRL-1660), as described previously [31].

Statistical analysis: Due to serological cross-reactivity among alphaviruses, we interpreted antibodies detected through IFA and ELISA against CHIKV to be alphavirus antibodies. To analyze the prevalence of alphavirus antibodies on PRNT, we excluded 20 samples that were positive for CHIKV IgG antibodies on IFA or CHIKV-IgM antibodies on ELISA but had not been tested on PRNT due to insufficient sample volume. We analyzed data using IBM SPSS Statistics (Version 22, IBM Corporation, Armonk, NY, USA). We tested for associations between demographic or clinical characteristics and alphavirus seropositivity using Fisher’s Exact Test (FET) with the level of significance set at <0.05 and odds ratios (OR) at a confidence interval (CI) of 95%.

## 3. Results

### 3.1. Participant Characteristics

In this study, a total of 557 samples were analyzed including 326 serum samples collected in Taita-Taveta in 2016, and 231 plasma samples collected in Kibera in 2017. Participant characteristics were reported in a previous study on flavivirus infection [31] and are summarized in Table 1.

### 3.2. CHIKV IgG IFA and IgM ELISA Antibody Assay Results

Out of 557 serum/plasma samples that were tested for CHIKV IgG antibodies on IFA, 49 (8.8%) were positive. IgG seroprevalence was 6.7% in samples from Taita-Taveta and 11.7% in samples from Kibera; *p* = 0.049 (FET).

Out of 553 serum/plasma samples that were tested for CHIKV IgM antibodies on ELISA, 33 (5.9%) were positive; 5.5% and 6.6% IgM seropositivity for Taita-Taveta and Kibera, respectively. The difference was not statistically significant; *p* = 0.59 FET. Four samples were positive for both CHIKV IgM and IgG.

### 3.3. Plaque Reduction Neutralization Assay Results

The 69 samples were tested on PRNT for CHIKV, ONNV, and SINV neutralizing antibodies. Of these 69 samples, 31 were positive for CHIKV IgG (IFA), 23 were positive for CHIKV IgM (ELISA), 4 were positive for both CHIKV IgG and IgM, and 11 were negative for CHIKV antibodies on IFA/ELISA. Then, 24 out of 31 (77%) of tested CHIKV IgG IFA positive samples were confirmed by PRNT, showing high concordance between PRNT and CHIKV IgG IFA. Only 1 out of 24 CHIKV IgM ELISA samples was positive in PRNT showing poor concordance between CHIKV IgM ELISA and PRNT results. Three of four samples that had both CHIKV IgM and IgG antibodies also tested positive on PRNT. Then, 1 out of 11 samples that were negative for both IgG and IgM antibodies was positive on PRNT. This sample was from a 19-year-old patient with fever for one day. Overall, 30 samples were found positive in PRNT, most (25/30) had higher titers for ONNV compared to CHIKV (Supplementary Table S1). Overall, 15/30 samples were positive for ONNV, 1/30 for CHIKV and 14/30 for both CHIKV and ONNV. None of the tested samples were positive for SINV. See Table 2.

Due to insufficient volume, 20 samples that had either CHIKV IgG (14) or CHIKV IgM (6) were not tested by PRNT. When these 20 samples were excluded from the analysis, the prevalence of alphavirus IgM antibody verified by PRNT was 4/537 (0.7%); 3/228 (1.3%) in Kibera and 1/309 (0.3%) in Taita-Taveta. The prevalence of alphavirus IgG antibodies verified by PRNT was 28/537 (5.2%); 21/228 (9.2%) in Kibera, and 7/309 (2.3%) in Taita-Taveta.

### 3.4. Factors Associated with Alphavirus Seropositivity

CHIKV IgG antibody seroprevalence (by PRNT) was higher in Kibera (9.2%) than in Taita Taveta (2.3%); *p* < 0.001 (FET). The prevalence was also significantly higher in adults (9.5%) than in children (1.1%); *p* < 0.001 (FET). IgG seropositive patients were also more likely to report joint pain than seronegative patients (8.5% vs. 3.3%, *p* = 0.015, FET). CHIKV IgG seroprevalence was higher in patients who had a history of travel to western Kenya (11.3% vs. 4.4%, *p* = 0.032, FET), as shown in Table 3.

In Taita-Taveta, 54 patients had a history of travel within 30 days before sample collection. Over two-thirds of the patients (37/54, 69%) had traveled to the coast (mainly Mombasa County). In Kibera, 88 patients had a history of travel, 56/88 (64%) having traveled to western Kenya (mainly Kisumu, Kakamega and Siaya Counties). Patients in Kibera were more likely to report travel to western Kenya than those in Taita-Taveta (OR = 11.3, CI 5.4–23.4; *p* < 0.001). History of travel to any part of Kenya was not significantly associated with arbovirus infection in either children or adults but travel to western Kenya was associated with risk of infection. Travel to western Kenya was also significantly associated with CHIKV IgM ELISA seropositivity (15.6% vs. 4.7%, OR: 3.8 (CI 1.69–8.30); *p* = 0.002, FET).

## 4. Discussion

Alphaviruses, especially CHIKV and ONNV, are an important cause of febrile illness in eastern Africa and have the potential to cause large outbreaks. Distinguishing ONNV and CHIKV from patient samples requires molecular detection methods that are not often feasible in clinical settings. Due to this cross-reactivity and the fact that CHIKV and ONNV have been documented to co-circulate [4,5], the epidemiological situation and health impact of the individual viruses is currently unclear.

In this study, we examined febrile patients from non-outbreak settings in two geographical areas for exposure to alphaviruses using a set of serological tests. Only four patients (0.7%) were positive for IgM antibody on ELISA confirmed by PRNT. This indicates that acute alphavirus infection was low in the study population and/or patients were sampled before antibodies were detectable. The poor correlation between PRNT and IgM ELISA could also be because neutralizing CHIKV IgM starts to appear. The median duration of fever in patients in this study was 2 days whereas IgM and IgG antibodies are usually detected after ~3–8 days and 4–10 days, respectively [38,39]. One sample was repeatedly negative on CHIKV IgG IFA and IgM ELISA but positive on PRNT. This was a sample from a 19-year-old patient with fever for one day and it is possible that early IgM antibody response is not detected as sensitively in the IgM-ELISA as with PRNT. Alternatively, PRNT may have detected antibody response against an epitope not present in the IgM ELISA assay.

Given the low prevalence of acute alphavirus infection, the two study areas appear to have been largely spared from the CHIKV outbreaks that occurred in coastal Kenya and Mandera in 2016, and in Mombasa from late 2017 to mid-2018 [11,12], whose timing approximated with our study sampling in Taita-Taveta in 2016, and Kibera in 2017.

The serological findings in this study represent a relatively high IgG seroprevalence with low IgM and the alphavirus IgG antibody seroprevalence of 6.7% for Taita-Taveta and 11.6% for Kibera is significant considering that these locations have no history of recorded alphavirus outbreaks. Notably, alphavirus seropositivity was higher among participants with a history of travel to western Kenya where CHIKV and ONNV infection is common [40]. Patients from Kibera were more likely to have traveled to western Kenya than those in Taita-Taveta. Alphavirus IgG seroprevalence in Nairobi city was nearly two-fold that of the more rural Taita-Taveta, but lower when compared to reports from coastal and western Kenya where previous studies detected alphavirus IgG seroprevalence as high as 26% by ELISA [5,40]. Since the primary aim of the study was to investigate the viral causes of acute febrile illness, only recent travel history was documented. Thus, the effect of possible earlier travel history that may have been a predictor of existing IgG antibodies cannot be confirmed. This may be especially crucial in the study area of Kibera, Nairobi, where relatively high rates of migration and travel are known to occur. Vector distribution in the two areas also supports the contribution of travel to alphavirus infections in Nairobi. *Culex* mosquitoes are the most abundant mosquito genus in Nairobi with infrequent reports of *Aedes*, mainly in a few pockets of forested areas, and even less frequent reports of *Anopheles* mosquitoes. Taita-Taveta has more mosquito vector diversity with *Culex* mosquitoes been the most abundant, *Aedes* frequently reported, and *Anopheles* mosquitoes more frequent than in Nairobi [41,42,43,44].

The results from confirmatory PRNT testing showed that none of the patients had neutralizing antibodies against SINV and that most of the PRNT-positive samples preferentially neutralized ONNV over CHIKV. This may be due to actual ONNV infection or cross-reactivity of CHIKV antibodies with ONNV as has been reported before [37,45]. It could also be due to sequential infection with both viruses in the past. Although we did not detect any SINV antibodies in this study, previous studies have reported very low SINV seroprevalence in humans [4,27]. Additionally, some studies have confirmed the circulation of SINV in mosquito vectors in Kenya, especially along the Great Rift Valley lakes and coastal region, which are located along the flyways for migratory birds [25,46,47].

Seroprevalence of alphavirus IgG antibodies was higher in adults than children. This is expected since exposure occurs over time. Participants with exposure to alphaviruses were also more likely to report joint pains than those not exposed to alphaviruses. Alphaviruses are known to cause chronic joint pain that may last for months to years [47,48].

When viewed together with our previous report on flaviviruses on the same samples [31], none of the samples had acute infection with both alphaviruses and flaviviruses while two samples had IgG antibodies for both virus groups, indicating that the transmission patterns for alphaviruses and flaviviruses are different in the study areas.

One limitation in this study is that the performance of our CHIKV IgG IFA assay has not been evaluated against any commercial ELISA. It is an in-house assay that is readily available and easy to carry-out. We considered it a reliable low-cost option for screening a large number of samples, before confirmation with PRNT. In our experience, it is comparable to CHIKV ELISA and has been used for diagnosis [46]. Similar IFAs have also been shown to have similar accuracy to ELISA [49,50].

Although all confirmed recent alphavirus outbreaks in Kenya have been caused by CHIKV, the risk of ONNV outbreak exists as the virus may be circulating unnoticed in both urban and rural areas, as indicated by previous studies [4,28,30]. The detection of higher ONNV antibody titers than CHIKV is in line with a previous study in which PRNT was used to distinguish ONNV from CHIKV in endemic coastal Kenya [5]. It is known that both CHIKV and ONNV can exist in sylvatic cycles in Africa [6,51], but the factors affecting their emergence and outbreaks are currently not well described.

More ONNV and CHIKV typing data from different areas of Kenya are needed for building a better understanding of their distribution and separate impacts on human health, such as long-term sequelae of alphavirus-associated arthralgia. The current understanding of ONNV and its impact on human health may be underestimated.

## Figures and Tables

**Table 1:** Characteristics of study participants.

Parameter	Taita-Taveta	Kibera	Total (%)
***n***	326 (59%)	231 (41%)	557
**Gender (*n* = 547)**			
Male	146 (46%)	115 (50%)	261 (48%)
Female	172 (54%)	114 (50%)	286 (52%)
**Age (*n*= 546)**			
Mean (Standard deviation (SD))	26.8 (22.1)	16.2 (15.3)	22.4 (20.2)
Median (Interquartile range (IQR))	20.6 (39.5)	10.2 (24.4)	16.4 (33.6)
Range	6 months–85 years	2 months–77 years	2 months–85 years
**Age Groups (*n* = 545)**			
0–5 years	69 (22%)	87 (39%)	156 (29%)
5–17 years	81 (25%)	45 (20%)	126 (23%)
18 years and above	169 (53%)	94 (41%)	263 (48%)
**Education Level in adults (*n* = 245)**			
None	15 (10%)	0 (0%)	15 (6%)
Primary school	77 (50%)	27 (30%)	104 (42%)
Secondary school	29 (19%)	36 (40%)	65 (27%)
Tertiary level	34 (22%)	27 (30%)	61 (25%)
**History of travel (*n* = 552)**			
Any travel outside the study area	54 (17%)	88 (38%)	142 (26%)
Travel to Nyanza or western Kenya	9 (3%)	56 (24%)	65 (12%)
Travel to coastal Kenya	37 (11%)	2 (1%)	39 (7%)
**Contact with animals (*n* = 557)**			
Contact with goats	134 (41%)	25 (11%)	159 (29%)
Contact with cattle	96 (29%)	26 (11%)	122 (22%)
Contact with chicken	190 (58%)	43 (19%)	233 (42%)
Contact with cats	61 (19%)	87 (38%)	148 (27%)
Contact with rodents	217 (67%)	127 (55%)	344 (62%)
Contact with bats	94 (29%)	6 (3%)	100 (18%)
**Signs and symptoms (*n* = 557)**			
Body Temperature in °C (Mean/SD)	38.8 (0.56)	38.4 (0.67)	38.6 (0.63)
Median duration of fever (range)	2 (1–7) days	2 (1–14)	2 (1–14) days
Joint pain	139 (43%)	70 (30%)	209 (38%)
Myalgia	123 (38%)	65 (28%)	188 (34%)
Headache	53 (16%)	108 (47%)	161 (29%)
Cough	44 (13%)	95 (41%)	139 (25%)
Vomiting	53 (16%)	68 (29%)	121 (22%)
Diarrhoea	29 (9%)	47 (20%)	76 (14%)
Rash	18 (6%)	16 (7%)	34 (6%)

**Table 2:** Alphavirus plaque reduction neutralization test results.

Sample Characteristics	*n*	CHIKV and ONNV Positive	CHIKV Positive	ONNV Positive	SINV Positive	Positive for Any Alphavirus	Negative
CHIKV IgG positive (IFA)	31	12 (39%)	0 (0%)	13 (42%)	0 (0%)	25 (81%)	6 (19%)
CHIKV IgM positive (ELISA)	23	0 (0%)	1 (4%)	0 (0%)	0 (0%)	1 (4%)	22 (96%)
Both CHIKV IgG and IgM positive	4	2 (50%)	0 (0%)	1 (25%)	0 (0%)	3 (75%)	1 (25%)
Negative	11	0 (0%)	0 (0%)	1 (9%)	0 (0%)	1 (9%)	10 (91%)
**TOTAL**	**69**	**14 (20%)**	**1 (1%)**	**15 (22%)**	**0 (0%)**	**30 (43%)**	**39 (57%)**

**Table 3:** Association between patient characteristics and alphavirus IgG antibodies on Plaque Reduction Neutralization assay (PRNT).

Characteristic	Alphavirus IgG Antibody Positive (PRNT)	Odds Ratio (95% CI)	*p* Value (Fisher’s Exact Test)
Age Group			
Adults	24/253 (9.5%)	9.5 (2.83–31.96)	<0.001
Children (<18 years)	3/275 (1.1%)		
Gender			
Female	15/274 (5.5%)	1.1 (0.49–2.29)	1.000
Male	13/253 (5.1%)		
Facility Location			
Kibera	21/228 (9.2%)	4.3 (1.83–10.48)	<0.001
Taita-Taveta	7/309 (2.3%)		
History of Travel			
Yes	9/137 (6.6%)	1.5 (0.64–3.36)	0.369
No	18/395 (4.6%)		
Travel to Western Kenya			
Yes	7/62 (11.3%)	2.8 (1.12–6.77)	0.032
No	21/475 (4.4%)		
Travel to Coastal Kenya			
Yes	1/38 (2.6%)	0.5 (0.06–3.58)	0.712
No	27/499 (5.4%)		
Joint Pain			
Yes	17/201 (8.5%)	2.7 (1.25–5.95)	0.015
No	11/336 (3.3%)		
Myalgia			
Yes	12/180 (6.7%)	1.5 (0.70–3.29)	0.307
No	16/357 (4.5%)		
Headache			
Yes	16/157 (10.2%)	3.5 (1.61–7.54)	0.002
No	12/380 (3.2%)		
Cough			
Yes	5/136 (3.7%)	0.6 (0.23–1.68)	0.503
No	25/401 (6.2%)		
Contact with goats			
Yes	4/148 (2.7%)	0.4 (0.14–1.24)	0.130
No	24/389 (6.2%)		
Contact with cattle			
Yes	4/117 (3.4%)	0.6 (0.20–1.72)	0.480
No	24/420 (5.7%)		
Contact with rodents			
Yes	15/331 (4.5%)	0.7 (0.33–1.51)	0.426
No	13/206 (6.3%)		
Contact with bats			
Yes	3/91 (3.3%)	0.6 (0.17–1.94)	0.450
No	25/446 (5.6%)		
Any animal contact			
Yes	19/419 (4.5%)	0.6 (0.25–1.31)	0.238
No	9/118 (7.6%)		

## Data Availability

The data presented in this study are available on request from the corresponding author.

## Supplementary Materials

The following supporting information can be downloaded at: https://www.mdpi.com/article/10.3390/v14061286/s1, Table S1: Characteristics of samples that tested positive for alphavirus antibodies on Plaque Reduction Neutralization Tests.
